# Association between leukocyte subpopulations and hematoma expansion after spontaneous intracerebral hemorrhage: A retrospective cohort study

**DOI:** 10.3389/fneur.2022.992851

**Published:** 2022-09-06

**Authors:** Jiao Qin, Haihua Wei, Yuling Liu, Lixin Du, Jun Xia

**Affiliations:** ^1^Department of Radiology, Shenzhen Longhua District Central Hospital, Shenzhen, China; ^2^Guangzhou Medical University, Guangzhou, China; ^3^Department of Nuclear Medicine, The First People's Hospital of Foshan, Foshan, China; ^4^Department of Radiology, Shenzhen Futian District Second People's Hospital, Shenzhen, China; ^5^Department of Radiology, Shenzhen Second People's Hospital, The First Affiliated Hospital of Shenzhen University, Shenzhen University, Shenzhen, China

**Keywords:** hematoma expansion, intracerebral hemorrhage, leukocyte, monocyte, neutrophil

## Abstract

**Aims:**

To verify the association between leukocyte subpopulations and hematoma expansion (HE) determined by two definitions in Chinese individuals who experienced spontaneous intracerebral hemorrhage.

**Methods:**

We enrolled 471 patients. The 1/2ABC formula was used to gauge hematoma volume. The outcome was whether HE appeared within 72 h. We used Definition 1 (volume increase ≥6 mL or 33%) and Definition 2 (volume increase ≥12.5 mL or 33%) to define HE, respectively. Binary logistic regression analysis was used to assess the association between leukocyte subpopulations and HE. For statistically significant leukocyte subpopulations, we also performed subgroup analyses to assess differences between subgroups.

**Results:**

Among 471 patients, 131 (27.81%) and 116 (24.63%) patients experienced HE based on Definition 1 and Definition 2, respectively. After adjusting for confounding factors, elevated monocyte count was associated with a higher risk of HE-Definition 1 [adjusted odds ratio (aOR) 2.45, 95% confidence interval (CI) 1.02–5.88, *P* = 0.0450] and HE-Definition 2 (aOR 2.54, 95% CI 1.04–6.20, *P* = 0.0399). Additionally, we compared the results before and after adjusting for coagulation parameters. Monocyte count was significantly correlated with HE only after adjusting for coagulation parameters. Increased neutrophil count was associated with a lower risk of HE-Definition 1 (aOR 0.91, 95% CI 0.84–1.00, *P* = 0.0463). No correlations were observed between lymphocyte and leukocyte counts and HE (*P* > 0.05), and no subgroup interactions were observed (interaction *P* > 0.05).

**Conclusion:**

A higher monocyte count is associated with a higher HE risk regardless of the two definitions, after excluding the influence of the coagulation parameters, which facilitates risk stratification. Moreover, an increased neutrophil count is associated with a decreased risk of HE in the context of HE-Definition 1, which reflects the importance of standardizing the definition of HE.

## Introduction

The number of deaths caused by stroke continues to increase globally, particularly in developing countries. Intracerebral hemorrhage (ICH) accounts for 10–15% of stroke cases and has the highest mortality rate (1). Accordingly, finding appropriate treatments for ICH is imperative. Early hematoma expansion (HE) after ICH is not only a potentially modifiable predictor of patient outcomes but also a promising therapeutic target (2–5). However, uncertainty regarding the risk factors for HE and the lack of specific predictors have led to the high disability and mortality related to ICH.

ICH is the rupture of diseased blood vessels and is characterized by the flow of blood into the brain parenchyma (5, 6). Once blood enters this region, the inflammatory response is activated (7). Within minutes of ICH onset, microglial cells are activated, marking the start of a potent inflammatory cascade (7, 8). Activated microglial cells initially exert their neuroprotective effects; however, hyperactive microglial cells release various cytokines and chemokines that promote the infiltration of peripheral leukocytes into the brain (7). Leukocytes infiltrating the hemorrhagic brain can produce reactive oxygen species, release pro-inflammatory mediators, and promote the breakdown of the blood–brain barrier (BBB), thereby aggravating ICH brain injury (7, 8). Previous studies reported that different leukocyte subpopulations have various effects on HE after ICH (9–11). However, to date, systematic clinical studies on the association of leukocytes and their subpopulations with HE among the Chinese population are lacking. Accordingly, this study aimed to retrospectively observe the association between leukocytes and their subpopulations with HE after spontaneous ICH.

## Methods

### Participant selection

We consecutively enrolled patients diagnosed with ICH using non-contrast computed tomography (NCCT) at Shenzhen Longhua District Central Hospital from May 2015 to November 2021. The inclusion criteria were as follows: (1) a diagnosis of spontaneous ICH detected by NCCT, (2) age at least 18 years old, and (3) leukocyte count obtained within 24 h of admission. In contrast, the exclusion criteria were as follows: (1) all secondary cerebral hemorrhage, including brain tumors, arteriovenous malformations, venous thrombosis, subarachnoid hemorrhage, thrombolysis-induced hemorrhage, and all trauma-related intracranial hemorrhage; (2) baseline NCCT and follow-up NCCT image artifacts significantly affecting volume measurement; and (3) hematoma surgery (including craniotomy and ventricular drainage) before follow-up NCCT.

**Figure 1:**
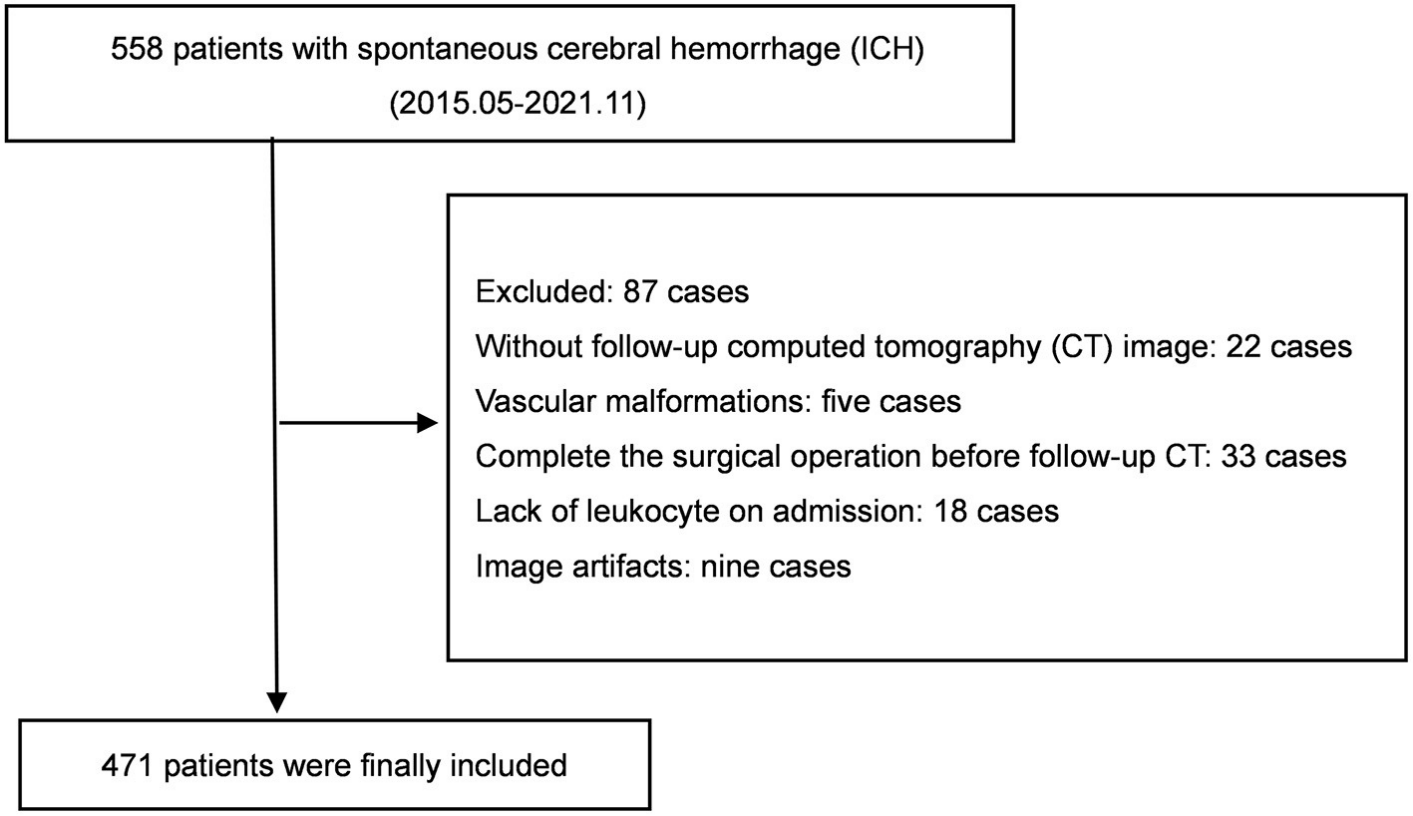
Flowchart of cohort selection.

We initially enrolled 558 patients with spontaneous ICH. Of these, 87 patients were excluded according to the following criteria: 22 patients whose follow-up NCCT images were missing, 33 who had undergone surgery before follow-up NCCT, 18 who exhibited leukocyte deficiency on admission, five who manifested vascular malformations, and nine whose images presented image artifacts. Ultimately, 471 patients were included (Figure 1).

This study was approved by the Ethical Review Committee of Shenzhen Longhua District Central Hospital (approval number: 2022-012-01). The need for informed consent was waived due to the study's retrospective design.

### Clinical data acquisition

We collected basic patient information by retrospectively reviewing outpatient medical records, including age, sex, systolic blood pressure at admission, and time from onset to admission. We combined the outpatients' medical records with those of inpatients to determine the history of hypertension, diabetes, hyperlipidemia, mannitol use, and the Glasgow Coma Scale score.

### Laboratory indicator collection

All laboratory indicators were assessed within 24 h of admission; when the patient had multiple test results, the first test result was preferentially selected for analysis. The laboratory indicators collected comprised thrombin time (TT); activated partial thromboplastin time (APTT); plasma fibrinogen determination (Fib); international standardized ratio; prothrombin time; red blood cell counts; hemoglobin levels; platelet counts; and leukocyte, lymphocyte, neutrophil, and monocyte counts. We also evaluated electrolyte levels that may impact ICH (potassium, chloride, and phosphorus levels) (12–14).

### Imaging

The NCCT scan used axial technology and 5-mm thick slices at 120–140 kV (peak) and 10–500 mA. The first NCCT image obtained at admission was selected as the baseline image, whereas the last NCCT image obtained within 72 h was selected as the follow-up image.

Imaging assessments were performed independently by two attending radiologists, and investigators were blinded to all clinical and laboratory variables. When inconsistencies were observed, solutions were obtained through consultation and discussion with senior physicians. First, we determined the location of the ICH (deep, lobar, and infratentorial) and its penetration of the ventricular system on baseline NCCT images. Notably, we included ICH of the basal nucleus, internal capsule, external capsule, insula, and thalamus in deep ICH. Second, the two attending radiologists independently used the 1/2 ABC formula to gauge the hematoma volume on the baseline NCCT image and follow-up NCCT image. Specifically, we regarded the NCCT maximum bleeding diameter as A, the diameter perpendicular to A as B, and the approximation of the NCCT bleeding layer multiplied by the layer thickness as C (15). To avoid subjective differences, we measured follow-up hematoma volumes after completing baseline hematoma volume measurements for all patients and then averaged the two pre- and post-volume measurements observed by the two radiologists. Finally, we subtracted the average baseline hematoma volume from the average follow-up hematoma volume to obtain the difference. Accordingly, dividing the difference by the average baseline hematoma volume provided the percent change in hematoma volume.

### Outcome measures

The study outcome was HE, which referred to the absolute and relative increase in hematoma volume at follow-up CT compared with the baseline hematoma volume. We used two methods recognized by researchers to define HE (16–21). For the convenience of expression, we termed these as HE-Definition 1 (absolute volume increase ≥6 mL or 33% relative volume increase) and HE-Definition 2 (absolute volume increase ≥12.5 mL or 33% relative volume increase).

### Statistical analysis

Baseline characteristic variables are presented as mean ± standard deviation or median and interquartile range based on the consistency of the data with a normal distribution. Categorical variables are indicated as percentages. Depending on the type of data, one-way analysis of variance, the χ^2^ test, or the Kruskal–Wallis *H*-test, was used to describe the normal distribution, categorical variables, or skewed distribution, respectively.

We used univariate analysis and a binary logistic regression model to assess the relationship between leukocytes and their subpopulations on HE. In the binary logistic regression model, the odds ratios (ORs) and 95% confidence intervals (CIs) of the unadjusted Model, Model I, and Model II were calculated. To control for confounders in Model I, we adjusted for sex, age, location, baseline hematoma volume, time from onset to admission, history of hypertension, hyperlipidemia, and the Glasgow Coma Scale score. To observe the effect of coagulation parameters on the results in Model II, we additionally adjusted for coagulation parameters (TT, Fib, and APTT) with *P* < 0.1 based on Model I.

We also performed subgroup analyses based on demographic characteristics (age <60 vs. ≥60 years; sex); clinical severity (Glasgow Coma Scale score <8 vs. ≥8 points; baseline hematoma volume <30 vs. ≥30 mL; systolic pressure <180 vs. ≥180 mmHg); and medical history (time from onset to admission <6 vs. ≥6 h). We evaluated the effect of subgroups on the relationship between monocyte or neutrophil counts and HE by adding an interaction term to the models. Data are presented as ORs and 95% CIs. Binary logistic regression was used to examine associations between neutrophil or monocyte counts and HE in different subgroups, and *P*-values for the interaction were recorded.

A two-tailed *P*-value < 0.05 was considered as statistically significant in all analyses. The analyses were performed with R software, version 3.6.1 (R Foundation for Statistical Computing, Vienna, Austria); Empower, version 2.21 (X&Y Solutions, Boston, MA); and GraphPad Prism, version 9.0.0 (GraphPad Software, San Diego, CA).

## Results

The average age of the study participants was 52.62 ± 12.28 years, and there were 327 men (69.43%) (Table 1). According to HE-Definition 1 and HE-Definition 2, 131 (27.81%) and 116 (24.63%) patients experienced HE, respectively.

**Table 1:** Baseline characteristics of the included patients.

**Variables**	**All cohort (*n* = 471)**
Age, mean (SD), years	52.62 (12.28)
Baseline volume, mean (SD), mL	10.50 (5.10–22.87)
TT, mean (SD), s	18.09 (3.84)
Fib, mean (SD), g/L	2.92 (0.89)
APTT, mean (SD), s	24.08 (4.86)
INR, mean (SD)	0.96 (0.18)
PT, mean (SD), s	10.94 (2.13)
Total leukocyte count, mean (SD), × 10^9^/L	9.42 (3.45)
Neutrophil count, median (IQR), × 10^9^/L	5.58 (4.25–7.73)
Lymphocyte count, median (IQR), × 109/L	1.96 (1.34–2.89)
Monocyte count, median (IQR), × 109/L	0.45 (0.31–0.59)
Red blood cell, mean (SD) × 10^12^/L	4.82 (0.64)
Hemoglobin, mean (SD), g/L	142.38 (18.91)
Platelet, mean (SD), × 10^9^/L	225.91 (61.80)
Potassium, median (IQR), mmol/L	3.74 (3.46–4.11)
Chlorine, mean (SD), mmol/L	104.25 (4.05)
Phosphorus, mean (SD), mmol/L	1.03 (0.24)
Time from onset to admission, median (IQR), hours	1.00 (0.67–4.00)
Systolic pressure, mean (SD), mmHg	178.78 (32.30)
Glasgow coma scale score, mean (SD)	12.56 (3.24)
**Sex**, ***n*** **(%)**	
Male	327 (69.43)
Female	144 (30.57)
**Baseline intraventricular hemorrhage**, ***n*** **(%)**	
No	350 (75.11)
Yes	116 (24.89)
**Location**, ***n*** **(%)**	
Deep	355 (76.18)
Lobar	46 (9.87)
Infratentorial	65 (13.95)
**Use mannitol before repeat CT**, ***n*** **(%)**	
No	246 (53.59)
Yes	213 (46.41)
**History of hypertension**, ***n*** **(%)**	
No	76 (16.89)
Yes	374 (83.11)
**History of diabetes**, ***n*** **(%)**	
No	409 (91.29)
Yes	39 (8.71)
**Hyperlipidemia**, ***n*** **(%)**	
No	411 (91.74)
Yes	37 (8.26)
**HE-Definition 1**, ***n*** **(%)**	
No	340 (72.19)
Yes	131 (27.81)
**HE-Definition 2**, ***n*** **(%)**	
No	355 (75.37)
Yes	116 (24.63)

SD, standard deviation; TT, thrombin time; Fib, plasma fibrinogen assay; APTT, activated partial thromboplastin time; INR, international normalized ratio; PT, prothrombin time; IQR, interquartile range; CT, computed tomography; HE-Definition 1, hematoma expansion (relative volume ≥33% or absolute volume ≥6 mL); HE-Definition 2, hematoma expansion (relative volume ≥33% or absolute volume ≥12.5 mL).

Univariate analysis (Table 2) showed that baseline volume, TT, Fib, neutrophil count, lymphocyte count, history of hypertension, hyperlipidemia, and Glasgow Coma Scale score were associated with HE according to both definitions (*P* < 0.05). APTT, monocyte count, and red blood cells were only associated with HE-Definition 2 (*P* < 0.05).

**Table 2:** Univariate analysis of hematoma expansion (HE) based on two definitions.

	**HE-definition 1**		**HE-definition 2**	
	**OR, 95% CI**	***P*** **-value**	**OR, 95% CI**	***P*** **-value**
**Sex**				
Male	ref		ref	
Female	0.86 (0.55–1.34)	0.4961	0.87 (0.55–1.39)	0.5673
Age (years)	0.99 (0.97–1.01)	0.2483	0.99 (0.97–1.00)	0.1289
**Baseline intraventricular hemorrhage**				
No	ref		ref	
Yes	1.10 (0.69–1.74)	0.6954	0.96 (0.59–1.57)	0.8763
Baseline volume (mL)	1.03 (1.02–1.04)	<0.0001^*^	1.02 (1.01–1.03)	0.0014^*^
**Location**				
Deep	Ref		ref	
Lobar	0.91 (0.45–1.83)	0.7974	0.87 (0.41–1.82)	0.7102
Infratentorial	1.15 (0.65–2.04)	0.6357	1.39 (0.78–2.48)	0.2656
TT (s)	1.27 (1.09–1.48)	0.0022^*^	1.23 (1.05–1.44)	0.0105^*^
Fib (g/L)	0.69 (0.53–0.90)	0.0068^*^	0.71 (0.54–0.94)	0.0165^*^
APTT (s)	1.04 (1.00–1.09)	0.0749	1.05 (1.00–1.10)	0.0298^*^
INR	1.39 (0.46–4.14)	0.5585	1.44 (0.47–4.39)	0.5215
PT (s)	1.04 (0.96–1.14)	0.3337	1.05 (0.96–1.14)	0.3198
Total leukocyte count (× 10^9^/L)	0.99 (0.93–1.05)	0.6972	1.00 (0.94–1.06)	0.9294
Neutrophil count (× 10^9^/L)	0.93 (0.87–0.99)	0.0294^*^	0.93 (0.87–1.00)	0.0493^*^
Lymphocyte count (× 10^9^/L)	1.21 (1.06–1.39)	0.0062^*^	1.22 (1.06–1.41)	0.0053^*^
Monocyte count (× 10^9^/L)	1.76 (0.91–3.42)	0.0952	2.08 (1.05–4.14)	0.0368^*^
Red blood cell (× 10^12^/L)	1.37 (0.99–1.88)	0.0566	1.43 (1.03–2.00)	0.0348^*^
Hemoglobin (g/L)	1.01 (1.00–1.02)	0.2120	1.01 (1.00–1.02)	0.1255
Platelet (× 10^9^/L)	1.00 (1.00, 1.00)	0.6290	1.00 (1.00–1.00)	0.5044
Potassium (mmol/L)	1.00 (0.99–1.00)	0.1581	1.00 (0.99–1.00)	0.1788
Chlorine (mmol/L)	0.97 (0.92–1.02)	0.2363	0.98 (0.93–1.04)	0.4882
Phosphorus (mmol/L)	0.41 (0.04–4.35)	0.4625	0.56 (0.05–6.05)	0.6291
Time from onset to admission (hours)	0.98 (0.97–1.00)	0.0551	0.99 (0.97–1.00)	0.1153
**Mannitol use before follow-up CT**				
No	ref		Ref	
Yes	0.71 (0.47–1.08)	0.1071	0.73 (0.47–1.11)	0.1420
Systolic blood pressure (mmHg)	1.00 (0.99–1.00)	0.2977	1.00 (0.99–1.00)	0.5759
**History of hypertension (mmHg)**				
No	ref		Ref	
Yes	2.93 (1.45–5.90)	0.0026^*^	3.75 (1.67–8.42)	0.0014^*^
**History of diabetes**				
No	ref		Ref	
Yes	0.88 (0.42–1.87)	0.7420	0.91 (0.42–1.99)	0.8226
**Hyperlipidemia**				
No	ref		Ref	
Yes	0.30 (0.10–0.86)	0.0249^*^	0.26 (0.08–0.85)	0.0266^*^
Glasgow Coma Scale score	0.87 (0.82–0.93)	<0.0001^*^	0.88 (0.83–0.94)	0.0002^*^

CI, confidence interval; OR, odds ratio; TT, thrombin time; Fib, plasma fibrinogen assay; APTT, activated partial thromboplastin time; INR, international normalized ratio; PT, prothrombin time; CT, computed tomography; HE-Definition 1, hematoma expansion (relative volume ≥33% or absolute volume ≥6 mL); HE-Definition 2, hematoma expansion (relative volume ≥33% or/and absolute volume ≥12.5 mL). Statistically significant values are in asterisks. Variables are presented as *n* (%) for nominal data and as mean ± standard deviation or median (interquartile range) for continuous data.

The

* symbol indicates the statistically significant values.

Table 3 showed the results of binary logistic regression analysis. In the unadjusted Model, neutrophil count was associated with HE-Definition 1 (OR: 0.93, 95% CI: 0.87–1.00, *P* = 0.0294) and HE-Definition 2 (OR: 0.93, 95% CI: 0.87–1.00, *P* = 0.0493); similarly, lymphocyte count was associated with HE-Definition 1 (OR: 1.21, 95% CI: 1.06–1.39, *P* = 0.0062) and HE-Definition 2 (OR: 1.22, 95% CI: 1.06–1.41, *P* = 0.0053). Monocyte count was not associated with HE-Definition 1 (OR: 1.76, 95% CI: 0.91–3.42, *P* = 0.0952) but was associated with HE-Definition 2 (OR: 2.08, 95% CI: 1.05–4.14, *P* = 0.0368). Meanwhile, leukocyte count was not associated with HE-Definition 1 (OR: 0.99, 95% CI: 0.93–1.05, *P* = 0.6972) or HE-Definition 2 (OR: 1.00, 95% CI: 0.94–1.06, *P* = 0.9295).

**Table 3:** Binary logistic regression of the relationship of leukocytes and their subpopulations with hematoma expansion (HE) based on two definitions.

	**HE-definition 1**			**HE-definition 2**		
**Exposure**	**Non-adjusted** **OR, 95% CI, P**	**Model I** **OR, 95% CI, P**	**Model II** **OR, 95% CI, P**	**Non-adjusted** **OR, 95% CI, P**	**Model I** **OR, 95% CI, P**	**Model II** **OR, 95% CI, P**
Total leukocyte count	0.99 (0.93–1.05) 0.6972	0.92 (0.85–1.00) 0.0411	0.97 (0.89–1.05) 0.4026	1.00 (0.94–1.06) 0.9295	0.93 (0.86–1.01) 0.0660	0.97 (0.89–1.06) 0.4841
Neutrophil count	0.93 (0.87–1.00) 0.0294	0.87 (0.80–0.95) 0.0015	0.91 (0.84–1.00) 0.0463	0.93 (0.87–1.00) 0.0493	0.88 (0.80–0.96) 0.0037	0.92 (0.84–1.01) 0.0774
Lymphocyte count	1.21 (1.06–1.39) 0.0062	1.14 (0.96–1.34) 0.1257	1.14 (0.96–1.34) 0.1414	1.22 (1.06–1.41) 0.0053	1.13 (0.95–1.33) 0.1598	1.12 (0.95–1.33) 0.1817
Monocyte count	1.76 (0.91–3.42) 0.0952	1.61 (0.75–3.44) 0.2178	2.45 (1.02–5.88) 0.0450	2.08 (1.05–4.14) 0.0368	1.73 (0.80–3.76) 0.1627	2.54 (1.04–6.20) 0.0399

OR, odds ratio; CI, confidence interval; HE-Definition 1, hematoma expansion (relative volume ≥33% or absolute volume ≥6 mL); HE-Definition 2, hematoma expansion (relative volume ≥33% or absolute volume ≥12.5 mL). Model I was adjusted for sex, age, location, baseline hematoma volume, time from onset to admission, history of hypertension, hyperlipidemia, and Glasgow Coma Scale score. Model II was adjusted for sex, age, location, baseline hematoma volume, time from onset to admission, history of hypertension, hyperlipidemia, Glasgow Coma Scale score, TT, Fib, and APTT.

In Model I, leukocyte count [adjusted OR (aOR): 0.92, 95% CI: 0.85–1.00, *P* = 0.0411] and neutrophil count (aOR: 0.87, 95% CI: 0.80–0.95, *P* = 0.0015) were associated with HE-Definition 1, whereas lymphocyte count (aOR: 1.14, 95% CI: 0.96–1.34, *P* = 0.1257) and monocyte count (aOR: 1.61, 95% CI: 0.75–3.44, *P* = 0.2178) were not significantly associated with HE-Definition 1. However, leukocyte count (aOR: 0.93, 95% CI: 0.86–1.01, *P* = 0.0660), lymphocyte count (aOR: 1.13, 95% CI: 0.95–1.33, *P* = 0.1598), and monocyte count (aOR: 1.73, 95% CI: 0.80–3.76, *P* = 0.1627) were not associated with HE-Definition 2, whereas neutrophil count was significantly associated with HE-Definition 2 (aOR: 0.88, 95% CI: 0.80–0.96, *P* = 0.0037).

**Figure 2:**
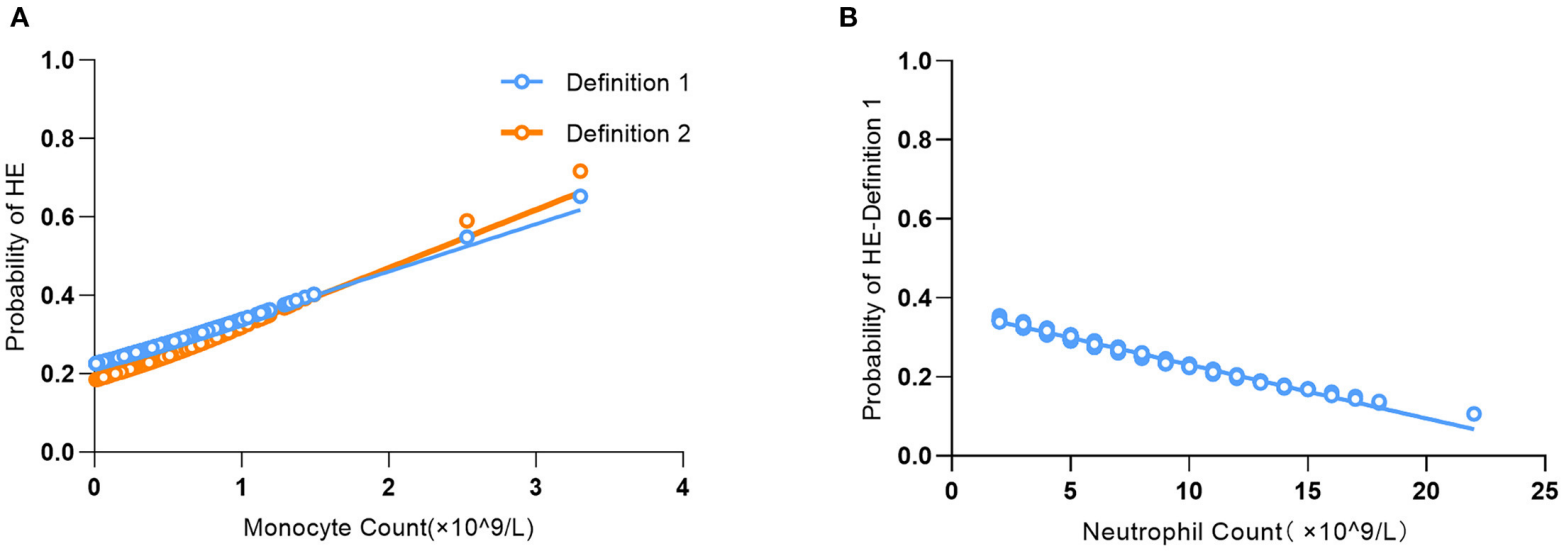
Association of monocyte count **(A)** and neutrophil count **(B)** with the probability of predicting hematoma expansion (HE).

In Model II, for every 1-unit increase in monocyte count, the risk of HE-Definition 1 increased by 1.45 times (aOR: 2.45, 95% CI: 1.02–5.88, *P* = 0.0450), and the risk of HE-Definition 2 increased by 1.54 times (aOR: 2.54, 95% CI: 1.04–6.20, *P* = 0.0399). Each unit increase in the neutrophil count was associated with a 9% reduction in the risk of HE-Definition 1 (aOR: 0.91, 95% CI: 0.84–1.00, *P* = 0.0463); however, it was not associated with HE-Definition 2 (aOR: 0.92, 95% CI: 0.84–1.01, *P* = 0.0774). The relationship between monocyte and neutrophil counts and HE is shown in Figure 2. Total leukocyte count and lymphocyte count were not significantly associated with HE-Definition 1 and HE-Definition 2 (all *P* > 0.05).

**Figure 3:**
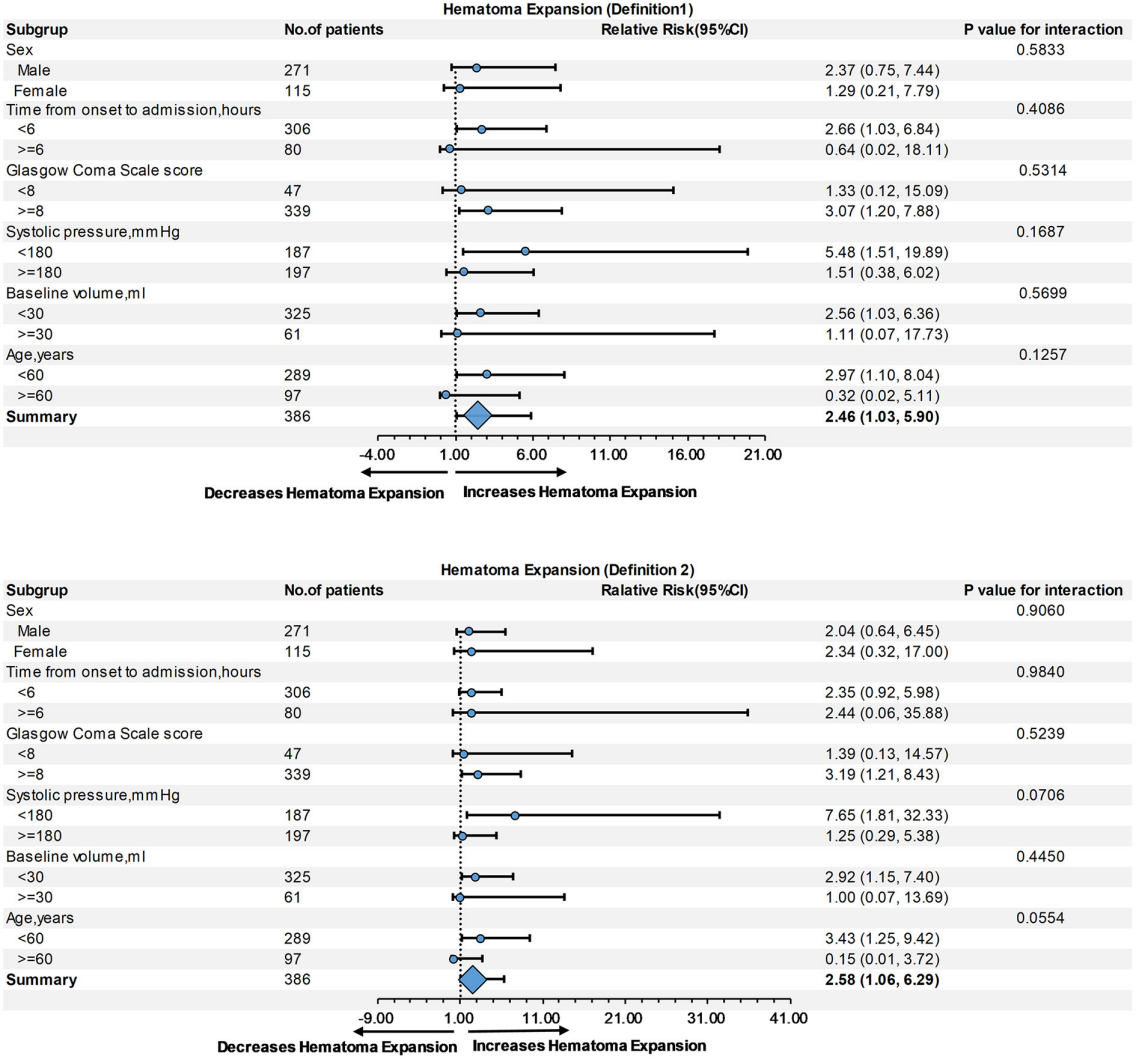
Subgroup analysis of the relationship of monocyte count and hematoma expansion (HE) according to Definitions 1 and 2.

**Figure 4:**
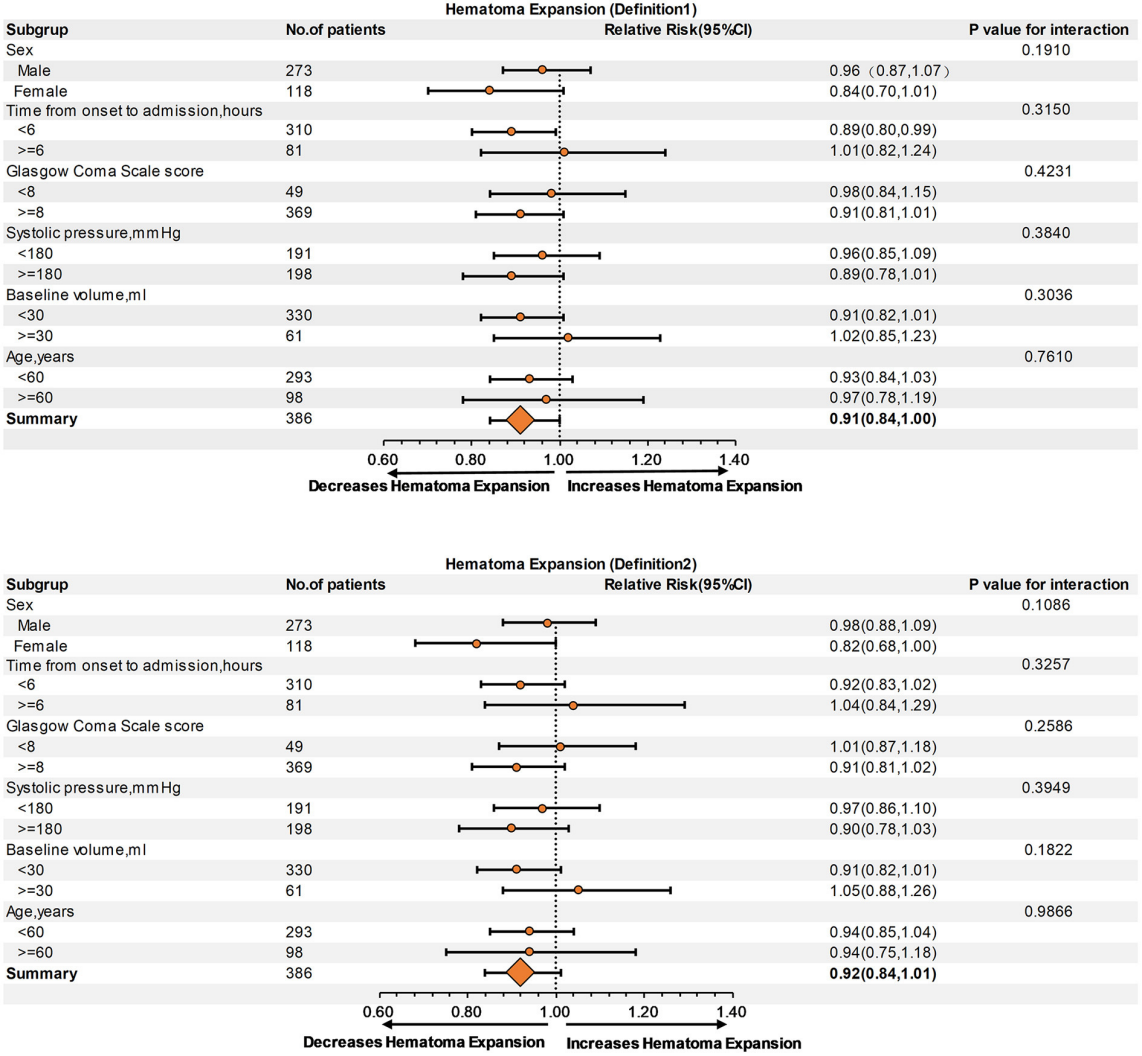
Subgroup analysis of the relationship of neutrophil count and hematoma expansion (HE) according to Definitions 1 and 2.

Subgroup analysis showed no interaction between age, sex, baseline hematoma volume, Glasgow Coma Scale score, systolic blood pressure, or time from onset to admission, regardless of HE-Definition 1 or HE-Definition 2 in the relationship of monocyte (Figure 3) and neutrophil counts (Figure 4) with HE (interaction *P* > 0.05).

## Discussion

Our findings suggest that an elevated monocyte count is related to a higher risk of HE regardless of the two definitions of HE, and the relationship between monocyte count and the risk of HE is independent of coagulation parameters. Moreover, an increased neutrophil count is associated with a decreased risk of HE in the context of HE-Definition 1, and this relationship is independent of coagulation parameters.

Morotti et al. previously reported a relationship between leukocyte count and HE in a population from Boston (10); the present results verify some of these results and expand them to include a Chinese population. The most significant similarities between the two studies are the positive correlation of monocyte count with HE and the lack of a relationship between lymphocyte count and HE. However, there are also two important differences. First, after adjusting for coagulation parameters, we found that the total leukocyte count was no longer associated with HE, and the relationship between monocyte count and HE was first presented; however, the relationship between neutrophil count and HE was not affected by coagulation parameters. These results suggest an interaction between leukocyte count and coagulation parameters, and different leukocyte subsets responded differently to coagulation parameters. Second, we only observed a relationship between neutrophil count and HE-Definition 1, which reflects the influence of the method used to define HE on the results.

Evidently, both definitions of HE resulted in consistent conclusions regarding the relationship between monocyte count and HE, thereby improving the reliability of our conclusions. Our results are consistent with a previous study, which suggested that the correlation between elevated monocyte levels and HE is mainly regulated by coagulation (10). Therefore, we compared the results before and after adjusting for the coagulation parameters (TT, Fib, and APTT). The evidence shows that the relationship between monocytes and the coagulation process is complex, with simultaneous procoagulant and anticoagulant properties. Under normal physiological regulations, the two are relatively balanced, and inflammatory stimulation can change the balance (22–24). Conversely, the expression of monocyte-derived tissue factors can promote thrombin and stable fibrinogen production (22, 24). In contrast, thrombomodulin on the surface of monocytes inhibits procoagulant activity, interferes with thrombus formation, and destabilizes fibrin (23); previous studies have used this mechanism to explain the positive correlation between monocyte count and HE (10, 25). However, our study showed that coagulation parameters had a great impact on the relationship between monocyte count and HE, but the coagulation parameters were not the main reason for high monocyte count being associated with increased HE risk, because only after adjusting these for these coagulation parameters that the monocyte count significantly correlated with HE. We speculate that this may be due to the predominance of coagulation characteristics of monocytes over anticoagulation characteristics within 72 h after ICH; however, this needs to be proven by future research. Besides, Mei et al. (26) reported that the proportion of monocytes in peripheral blood increased during the acute phase of ICH (6 h after onset). Another study also suggested that changes in monocytes may be closely related to the process of ICH (27). However, there was no interaction between the subgroups (time from onset to admission <6 h vs. ≥6 h) in our study. In summary, our study provides clinical evidence that higher monocyte count is associated with an increased risk of HE.

Our study shows that elevated neutrophil count reduces the risk of HE only for Definition 1, which is somewhat consistent with the findings of the Boston study (10). The mechanism may be related to the release of lactoferrin from neutrophils, prompting an increase in iron clearance (11) and the marked procoagulant properties of activated neutrophils (10). The association between neutrophil count and HE-Definition 2 was almost statistically significant (*P* = 0.07). This highlights the impact of using different methods of defining HE on the results. Since there is no consensus definition for HE yet, it is difficult to determine which definition of HE is closer to the actual clinical phenomena. We observed that neutrophil count might have less influence on HE than monocyte count because of the impact of the HE definition. Hence, when compared with other studies wherein Definition 1 was used for HE, neutrophil count was as valuable as other risk factors for HE (28).

Morotti et al. (10, 29) reported that leukocyte count might reduce HE by enhancing coagulation because of the interactions between leukocytes and platelets, endothelial cells, and coagulation factors. In our study, after adjusting for TT, Fib, and APTT, the leukocyte count was not associated with HE. This confirms that leukocytes limit HE by interacting with the coagulation process rather than directly affecting HE processes.

Preclinical studies have shown that certain lymphocyte subsets can disrupt the BBB by reducing the expression of claudin, destroying endothelial cells and their tight junctions, and mediating endothelial cell apoptosis (26, 30); hence, the disruption of the BBB contributes to HE (31). These results suggest that lymphocytes participate in the disruption of the BBB, which inevitably leads to HE. However, to the best of our knowledge, this hypothesis has not been confirmed in clinical studies. Our study showed that lymphocyte count was not significantly associated with HE either.

The following five points describe the strengths of our study: first, our study demonstrates that the earlier findings reported by Morotti et al. are also applicable in China. Second, we adopted two definitions for HE to improve the study's accuracy. Third, our findings apply to a wider population because we did not limit the time from onset to patient admission. Most previous studies only included patients with HE within 24 h from onset (21, 32), and a few studies expanded this to 48 h (10). Fourth, other Chinese studies are limited to the role of a single leukocyte type in ICH, which makes it difficult to exclude population differences when interpreting the results of different studies. However, our study is valuable for future research because of the simultaneous analysis of the role of the three leukocyte subsets in HE in the same population. Finally, leukocyte count is a readily available laboratory indicator during admission that can be obtained cost-effectively and is easily instituted, making our methodology and results easily translatable to clinical settings.

In summary, our study contributes to the risk stratification of HE in an acute clinical setting. For patients with a higher monocyte count and lower neutrophil count, physicians should shorten the time for NCCT revision and provide more attentive care. Furthermore, considering the diametrically opposed representations of monocytes and neutrophils in HE, physicians should be cautious of employing traditional holistic anti-inflammatory approaches for treating patients with ICH and consider individualizing the strengths of different subpopulations.

Regardless, our study had the following limitations. First, our study was limited by the retrospective nature of the cohort; therefore, some patients were excluded because of a lack of routine blood tests and follow-up NCCT. Second, the results cannot be extrapolated to patient groups excluded from the study. Third, we did not exclude other systemic diseases that might affect leukocytes and their subpopulations, which might slightly weaken our findings; however, we still obtained results that were somewhat similar to previous studies. Fourth, the 1/2ABC formula has been proven to overestimate the hematoma volume (33). Hence, even if the 1/2ABC formula was proved to be accurate in the change of hematoma volume (33), it might still affect the results. Fifth, our study lacked multi center data. Finally, our research population was limited to a Chinese population with certain geographical and ethnic restrictions, which may affect the generalizability of the results.

In conclusion, after excluding the influence of coagulation parameters, a higher monocyte count is associated with higher HE risks regardless of the two HE definitions, which facilitates risk stratification. Nevertheless, the underlying pathophysiological mechanisms requires further investigation. Moreover, an increased neutrophil count is associated with a decreased risk of HE in the context of HE-Definition 1, which reflects the importance of standardizing the definition of HE. More clinical studies are needed to explore a standard for HE.

## Data availability statement

Study data are not publicly available due to patient confidentiality. Study data are available from the corresponding author upon reasonable request.

## Ethics statement

The studies involving human participants were reviewed and approved by Ethical Review Committee of Shenzhen Longhua District Central Hospital (approval number: 2022-012-01). Written informed consent for participation was not required for this study in accordance with the national legislation and the institutional requirements.

## Author contributions

JQ and HW contributed to the research planning, data collection, and drafting of the manuscript. YL participated in data collection, contributed to the analysis of data, and assessed the risk of bias in the study. LD and JX provided professional guidance for this study and performed a final check of the manuscript. All authors contributed to the study described in this manuscript and approved the submitted version.

## Funding

This study was funded by the Natural Science Foundation of Guangdong Province (2020A1515010918), the Shenzhen Basic Development Project (JCYJ20190806164409040), the Shenzhen Natural Science Foundation (JCYJ20210324142404012), and the Key Laboratory of Neuroimaging of Longhua District.

## Conflict of interest

The authors declare that the research was conducted in the absence of any commercial or financial relationships that could be construed as a potential conflict of interest.

## Publisher's note

All claims expressed in this article are solely those of the authors and do not necessarily represent those of their affiliated organizations, or those of the publisher, the editors and the reviewers. Any product that may be evaluated in this article, or claim that may be made by its manufacturer, is not guaranteed or endorsed by the publisher.
